# Radiomics-based differentiation between glioblastoma and primary central nervous system lymphoma: CT vs MRI

**DOI:** 10.1186/s40644-026-01018-8

**Published:** 2026-03-16

**Authors:** Feifei Yu, Junhui Yuan, Xiaoye Lin, Feng Wang, Lan Yu, Shujie Yu, Youquan Zhu, Yang Song, Dairong Cao, Jieyun Chen, Zhen Xing

**Affiliations:** 1 Department of Radiology, The First Affiliated Hospital of Fujian Medical University, 20 Cha-Zhong Road, Fuzhou, Fujian 350005 China; 2 Department of Radiology, National Regional Medical Center, Binhai Campus of the First Affiliated Hospital, Fujian Medical University, Fuzhou, 350212 China; 3 Department of Medical Imaging, The Affiliated Cancer Hospital of Zhengzhou University & Henan Cancer Hospital, Zhengzhou, Henan Province 450008 China; 4 MR Research Collaboration Team, Siemens Healthineers Ltd., Shanghai, 200126 China; 5 Department of Radiology, Quanzhou First Hospital Affiliated to Fujian Medical University, 248-252 East Street, Licheng, Quanzhou, Fujian 362000 China; 6 Fujian Key Laboratory of Precision Medicine for Cancer, Department of Radiology, The First Affiliated Hospital, Fujian Medical University, Fuzhou, 350005 China; 7 Key Laboratory of Radiation Biology of Fujian Higher Education Institutions, The First Affiliated Hospital, Fujian Medical University, Fuzhou, 350005 China

**Keywords:** Glioblastoma, Primary central nervous system lymphoma, Radiomics, Computed tomography, Magnetic resonance imaging

## Abstract

**Background:**

To systematically evaluate and compare the diagnostic efficacy of radiomics models derived from noncontrast CT (NCCT) versus multiparametric MRI in differentiating glioblastoma (GBM) from primary central nervous system lymphoma (PCNSL).

**Methods:**

In this retrospective, multicenter study, 543 patients with pathologically confirmed GBM (*n* = 401) or PCNSL (*n* = 142) were divided into 3 cohorts. 1084 quantitative features were extracted from contrast-enhancing (CE) and non-enhancing (NE) regions across NCCT and five MRI sequences (T2WI, T1WI, ADC, FLAIR, and CE-T1WI). Feature selection employed ANOVA, Kruskal-Wallis test, and recursive feature elimination, followed by nested cross-validation (5-fold outer, 3-fold inner) to construct four machine learning classifiers: support vector machine, linear discriminant analysis, logistic regression, and decision tree. Model performance was rigorously assessed through AUC, accuracy, sensitivity, specificity with bootstrap-derived 95% confidence intervals. The Shapley Additive Explanation (SHAP) analysis was employed to explore the interpretability of models.

**Results:**

The CE-T1WI radiomics model demonstrated superior diagnostic capability, with its AUCs of train/internal test/external test in CE regions and NE regions were 0.962/0.963/0.907 and 0.966/0.892/0.867, respectively. Notably, the CT-based model was not significantly different from other MRI models except for CE-T1WI model. The AUCs of train/internal test/external test for CT model in CE and NE regions were 0.941/0.906/0.822 and 0.902/0.891 /0.782, respectively.

**Conclusions:**

Both NCCT and multiparametric MRI are valuable in identifying GBM and PCNSL. The CE-T1WI radiomics model has the best diagnostic efficacy.

**Supplementary Information:**

The online version contains supplementary material available at 10.1186/s40644-026-01018-8.

## Introduction

Glioblastoma (GBM) and primary central nervous system lymphoma (PCNSL) are the two most common types of primary malignant brain tumors with divergent therapies: GBM management relies on maximal safe resection followed by concurrent chemo- and radio-therapy whereas PCNSL typically requires biopsy-confirmed diagnosis prior to initiating high-dose methotrexate-based regimens with radiotherapy [1–3]. Thus, preoperative noninvasive and precise identification of GBM and PCNSL is crucial to optimize clinical decisions, prevent iatrogenic harm and improve patient outcomes.

Currently, GBM and PCNSL are primarily differentiated preoperatively using MRI. On conventional MRI, most GBM lesions show heterogeneous enhancement while most PCNSL lesions enhanced homogeneously [4, 5]. In terms of advanced MRI techniques, apparent diffusion coefficient (ADC) derived from diffusion-weighted imaging (DWI), rCBVs derived from dynamic sensitive contrast-enhanced perfusion-weighted imaging (DSC-PWI), and intratumoral susceptibility signals intensity (ITSS) measured by magnetic susceptibility-weighted imaging (SWI) were significantly lower in PCNSL patients than in GBM patients [6–9]. In addition, several studies have combined MRI with radiomics to extract and quantify the differences between PCNSL and GBM on MR images via high-throughput computation of high-dimensional image features [10, 11].

However, MRI is limited by contraindications (e.g., renal failure, implanted devices), long acquisition times, and resource disparities [12]. Of note, the first imaging examination a patient receives at the initial visit is usually noncontrast CT (NCCT). Yousem et al. demonstrated that 41% of CNS tumors in resource-limited settings are diagnosed primarily by CT imaging [13], especially in those cases involving contraindications to MRI or challenges with healthcare accessibility.

In the past, due to technological limitations, CT could provide less information, but with the advent of radiomics, which can provide more information from a dimensional perspective, the value of CT applications has increased significantly. CT-based radiomics has unique advantages over MRI: 1) Hounsfield unit (HU) values provide absolute quantification of tissue attenuation with excellent reproducibility across scanners; 2)the fast acquisition minimizes motion artifacts that can reduce feature stability [14]. Although Guang Lu et al. have demonstrated that CT radiomics exhibits high performance in the differential diagnosis of GBM and PCNSL [15], there remains a critical lack of research in this area and systematic comparative studies on the diagnostic efficacy between CT and MRI.

Therefore, the aim of this study was to explore the use of radiomic features extracted from NCCT to differentiate between GBM and PCNSL, and to compare the diagnostic performance of the CT model with various MRI models based on different parameters.

## Methods

The Institutional Review Board of The First Affiliated Hospital of Fujian Medical University (Center 1), Henan Cancer Hospital (Center 2) and Quanzhou First Hospital Affiliated to Fujian Medical University (Center 3) approved this retrospective study and waived the need for written informed consent.

### Study population

**Fig. 1:**
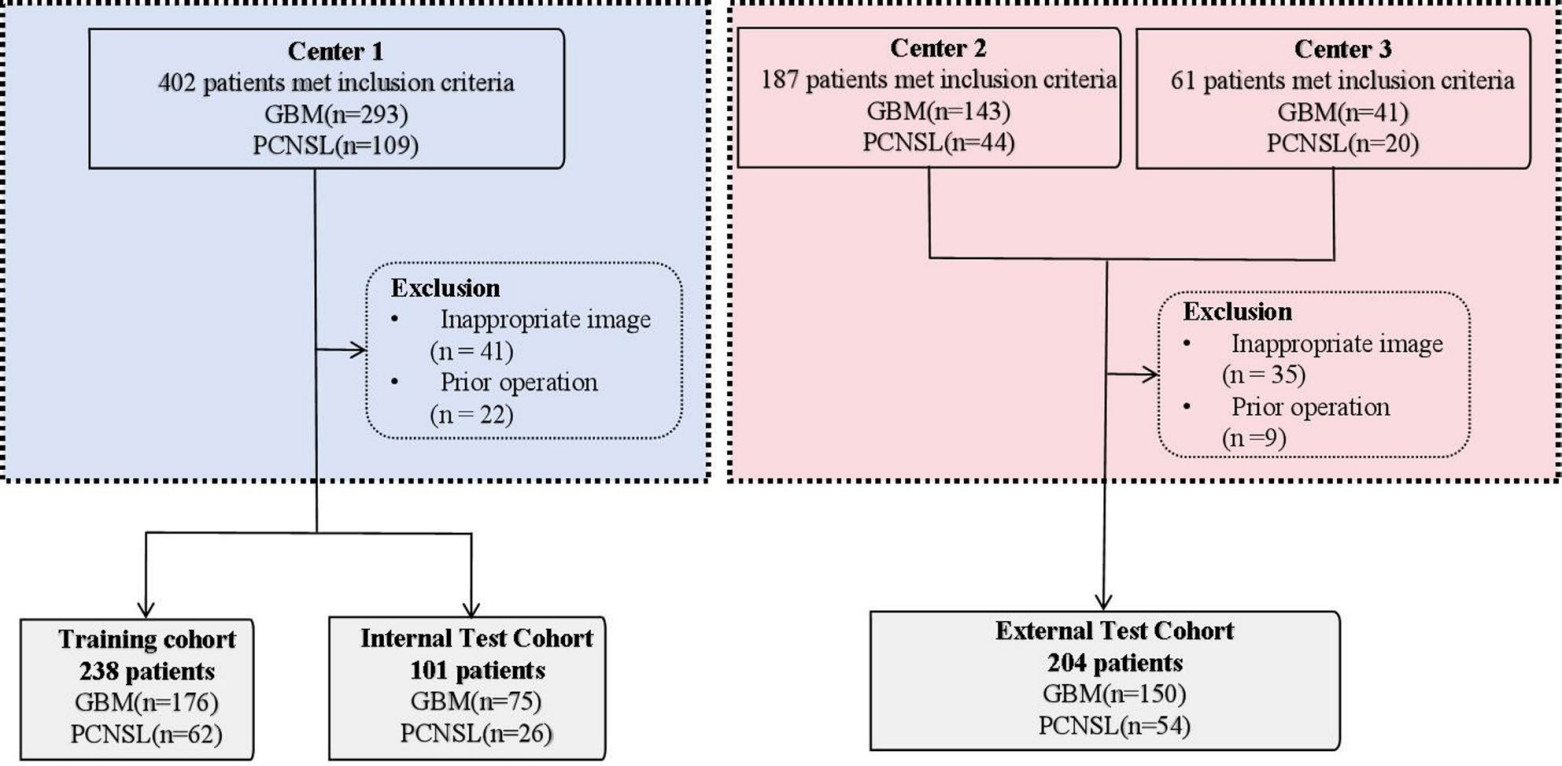
Flowchart of patient inclusion. A total of 339 patients were included at Center 1 and was randomly divided into a training cohort (238 patients) and an internal test cohort (101 patients) in a 7:3 ratio. Center 2 and Center 3 included 204 patients as an external test cohort. GBM glioblastoma; PCNSL primary central nervous system lymphoma

From January 2020 to December 2025, patients with pathologically confirmed diagnoses of GBM and PCNSL were screened at Center 1, Center 2 and Center 3. The inclusion criteria were as follows: (1) The patients were pathologically diagnosed with GBM or PCNSL; (2) The tumors were located in the cerebral cortex; (3) Preoperative NCCT and complete MRI images were available, including T2-weighted imaging (T2WI), T1-weighted imaging (T1WI), ADC, fluid-attenuated inversion recovery (FLAIR), and contrast-enhanced T1WI (CE-T1WI). The exclusion criteria were as follows: (1) The required images were incomplete or the image quality was insufficient for analysis; (2) There had been previous biopsies or surgeries. Patients from Center 1 were randomly allocated to the training cohort or internal test cohort, while patients from Center 2 and Center 3 formed the external test cohort. The patient enrollment process of this study is shown in Fig. 1.

### MR and CT imaging protocol

All centers adopted standardized imaging protocols for neurological MRI and CT examinations, with center-specific adjustments to device parameters to match hardware characteristics. Detailed information on scanner models, sequence parameters (e.g., field of view, slice thickness, matrix), and scanning modes for MRI (including T2WI, T1WI, FLAIR, CE-T1WI, and DWI sequences) and CT is provided in Additional file 1. Briefly, 3.0 Tesla MRI scanners were used across all centers for multiparametric MRI, and multi-detector spiral CT scanners were used for NCCT, with all devices operating in standard helical mode to minimize inter-device variability in image reconstruction parameters. ADC maps were automatically generated by respective MRI workstations for DWI sequences.

### Image registration and tumor segmentation

In this study, a multimodal image alignment strategy was used to ensure cross-sequence anatomical consistency. After format conversion of the raw DICOM data, a multistep alignment process was implemented based on the 3D Slicer platform (v4.11.0): firstly, CE-T1WI, T1WI, ADC, and FLAIR were aligned to the T2WI space by rigid-body transformation to generate the post-alignment images (rCE-T1WI, rT1, rADC, and rFLAIR). Subsequently, the cross-modal nonlinear alignment of CT images with rCE-T1WI was achieved by ITK-SNAP (v3.8.0) using the mutual information maximization algorithm to obtain the post-alignment CT (rCT). The quality of the alignment was verified by calculating the Normalized Mutual Information (NMI > 0.85) and visual inspection.

Tumor segmentation was performed within a consensus reading framework with center-specific optimization to balance accuracy and efficiency: For Center 1 and Center 2, two senior neuroradiologists independently manually delineated the contrast-enhancing (CE) and non-enhancing (NE) regions based on rCE-T1WI, T2WI, and rCT images. For Center 3, a semi-automatic deep learning–assisted workflow was adopted using 3D Slicer (v4.11.0) integrated with a pre-trained U-Net-based model to balance segmentation accuracy and efficiency: initial region masks were generated automatically by the model, followed by expert refinement of boundary inaccuracies. Discrepancies in < 5% of cases were resolved via consensus discussion. The reliability of segmentation results across all centers was quantitatively assessed by ICC (ICC > 0.90).

### Radiomics feature extraction

Based on the Imaging Biomarker Standardization Initiative (IBSI) version 2 guidelines, the open source software FeAture Explorer (v0.5.6) was used to perform a standardized feature extraction process. Image preprocessing included 1) voxel resampling to 1 × 1 × 1 mm³ isotropic space, 2) grayscale intensity normalization (Z-score), and 3) application of Laplacian of Gaussian filtering (σ = 1.0 mm) prior to non-texture feature extraction. A total of 1084 features were systematically extracted from each volume of interest (VOI) in seven categories: morphological features (14), first-order histogram features (17), Gray Level Cooccurrence Matrix (24), Gray Level Run Length Matrix (16), Gray Level Size Zone Matrix (16), Neighborhood Gray Tone Difference Matrix (5), and Gray Level Dependence Matrix (14). The feature extraction covered the CE and NE regions of five MRI sequences (T2WI, T1WI, CE-T1WI, FLAIR, and ADC) and NCCT, and 12 sub-feature sets were constructed (6 modalities × 2 anatomical partitions). The final generated feature matrix was calibrated for IBSI compatibility to ensure cross-platform reproducibility. To eliminate cross-center and cross-vendor batch effects, the ComBat algorithm was employed to harmonize the merged radiomic features: “center-vendor” was designated as the core batch variable, with diagnostic type serving as the covariate. The consistency of feature distributions before and after harmonization was verified using the Kolmogorov-Smirnov test (a *p*-value > 0.05 indicates no significant difference in feature distributions).

### Radiomics feature matrix pre-processing and feature selection

A two-stage normalization strategy was adopted for feature preprocessing: firstly, Z-score normalization was used to eliminate the magnitude difference, and then Mean-Variance Scaling was applied to enhance the linear divisibility of the feature space. To control multicollinearity, the Pearson correlation coefficient thresholding method (|r|>0.99) was used to eliminate redundant features and retain the maximum variance features.

Feature selection is often considered as a key part of the model building process. Its purpose is to select the most representative and discriminative subset of features from a large number of original features in order to reduce the complexity of the model and improve its performance. In this study, a hybrid strategy was used for feature selection. Features with significant between-group differences were screened by Kruskal-Wallis test (*p* < 0.05) and one-way analysis of variance (ANOVA, FDR-corrected) in the initial screening stage, and recursive feature elimination (RFE) combined with linear kernel of Support Vector Machine (SVM) was used in the fine screening stage for wraparound feature optimization. The optimal feature subset dimensions [1, 30] were determined by grid search, and the final number of features was determined based on a 5-fold cross-validated area under curve (AUC) plateau period. The plateau was defined by two criteria that must be met simultaneously: (1) after consecutively adding 2 feature dimensions, the increase in AUC relative to the initial peak was ≤ 2% for both additions; (2) the difference in AUC between the current feature subset and larger subsets was not statistically significant (*p* > 0.05) as assessed by the DeLong test. All calculationswas performed using FeAture Explore (V 0.5.8). To further improve the transparency of the feature selection process and the reproducibility of the study, the core supplementary data related to feature screening, stability verification, and preprocessing effects in this study are integrated into the same supplementary file (see Additional file 2) for details)

### Radiomic feature reproducibility analysis

To quantify the inter-observer and inter-time reproducibility of radiomic features, 30 cases (15 GBM, 15 PCNSL, from Center 1 and Center 2) were selected. Two senior neuroradiologists independently performed two rounds of tumor segmentation in a blinded manner at a 2-week interval. Radiomic features were extracted from NCCT and non-contrast MRI sequences (T1WI, T2WI, FLAIR, ADC, CE-T1WI).

A two-way mixed-effects model was used to calculate the ICC in accordance with the IBSI 2.0 guidelines. The classification criteria for ICC were defined as: ICC > 0.9 (excellent stability), 0.75–0.9 (good stability), and < 0.75 (insufficient stability). An independent samples t-test was applied to compare ICC differences across modalities, with a P-value < 0.05 considered statistically significant.

### Predictive model establishment

**Fig. 2:**
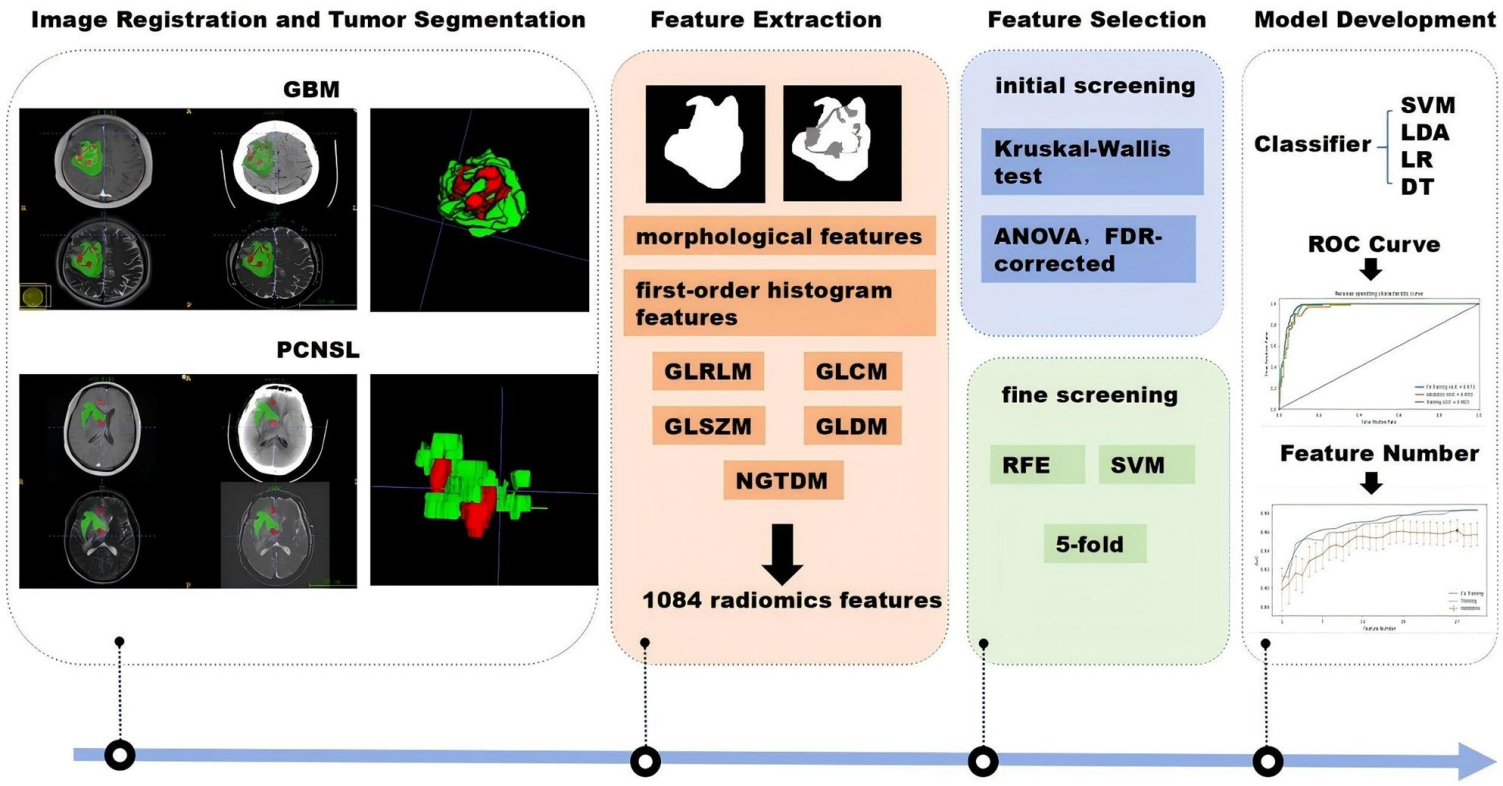
Workflow of model development. GBM glioblastoma; PCNSL primary central nervous system lymphoma; GLRLM, grayscale tour matrix; GLCM, grayscale covariance matrix; GLSZM, grayscale region size matrix; GLDM, grayscale dependency matrix; NGTDM, neighborhood grayscale difference matrix; ANOVA, analysis of variance; RFE, recursive feature elimination; SVM, support vector machine; LDA, linear discriminant analysis; LR, logistic regression; DT, decision tree

Stratified random sampling was used to divide the training and internal test cohort in a 7:3 ratio. Four types of classifiers were constructed based on scikit-learn (v1.0.2): SVM, linear discriminant analysis (LDA), logistic regression (LR), and decision tree (DT). Figure 2 presents the flowchart of the modeling method. Model training was performed using a nested cross-validation framework: outer 5-fold cross-validation to optimize hyperparameters and inner 3-fold cross-validation to assess generalization performance. Model performance was comprehensively evaluated by AUC, accuracy (ACC), sensitivity (SEN), and specificity (SPE), with reported 95% confidence intervals (Bootstrap method, *n* = 1000 resamples). Feature extraction and model training for the traditional machine learning models in this study were performed using FeAture Explore software (v0.5.8).

### Model interpretation

The SHAP (SHapley Additive exPlanation) method was employed to quantify the contribution weights of radiomic features across different modalities to diagnostic decisions, thereby enabling model interpretability analysis. Specifically, the mean absolute SHAP value (mean |SHAP|) was used to rank the importance of the features included in the model. Features with higher rankings exhibited higher mean |SHAP| values, which indicated that these features made a more significant predictive contribution to the model’s differentiation between GBM and PCNSL.

### Statistical analysis

All statistical analyses were performed using “Python 3.8.5 with scikit-learn”. Continuous variable normality was confirmed by the Shapiro-Wilk test (*p* > 0.05), and between-group comparisons were made using the independent samples t-test (normal distribution) or the Mann-Whitney U test (non-normal distribution). Categorical variables were compared using the χ² test or Fisher exact test (expected count < 5). All tests were two-sided and the significance level was set at α = 0.05. The DeLong test was used to assess whether the differences in AUC between models were statistically significant. *p* < 0.05 indicates statistical significance.

## Results

### Patient characteristic

A total of 543 patients were included in this study, including 401 with GBM and 142 with PCNSL. The training cohort consisted of 238 patients (176 GBM and 62 PCNSL), while the internal test cohort included 101 patients (75 GBM and 26 PCNSL). The external test cohort comprised 204 patients, including 150 with GBM and 54 with PCNSL. The baseline characteristics of the patients are shown in Table 1.

**Table 1:** Baseline characteristics of patients in three cohorts

		Training cohort	Internal test cohort	External test cohort
GBM		176	75	150
	Age* Sex(M : F)	62.8 ± 7.85 123:53	64.3 ± 8.12 60:15	53.4 ± 15.20 80:70
PCNSL		62	26	54
	Age* Sex(M : F)	65.2 ± 6.92 37:25	67.1 ± 7.01 13:13	60.2 ± 12.66 29:25

*Data are means ± standard deviations

### ICC-based feature reproducibility

The results showed that the feature stability of NCCT was generally comparable to that of non-contrast MRI sequences, with slight advantages in specific feature types, and detailed data are presented in Table 2 : The overall mean ICC of NCCT was 0.90 ± 0.02, showing no significant differences from CE-T1WI (0.89 ± 0.02) and ADC (0.88 ± 0.02) (*p* = 0.21 and 0.18, respectively), with only a slight difference from T1WI (0.85 ± 0.03, *p* = 0.04); Furthermore, the “Excellent Feature Rate” of NCCT (proportion of features with ICC > 0.9) reached 78.5%, which was higher than that of all non-contrast MRI sequences (65.2%–74.8%).

**Table 2:** Comparison of ICC values of radiomic features across modalities (± SD, *n* = 30)

Feature Category	NCCT	CE-T1WI	ADC	T2WI	FLAIR	T1WI
Morphological	0.93 ± 0.01	0.92 ± 0.01	0.91 ± 0.01	0.89 ± 0.02	0.88 ± 0.02	0.87 ± 0.02
First-order Histogram	0.91 ± 0.01	0.89 ± 0.01	0.90 ± 0.01	0.88 ± 0.02	0.87 ± 0.02	0.86 ± 0.02
Texture	0.86 ± 0.02	0.88 ± 0.02	0.87 ± 0.02	0.87 ± 0.02	0.85 ± 0.02	0.84 ± 0.02
Overall Mean	0.90 ± 0.02	0.89 ± 0.02	0.88 ± 0.02	0.87 ± 0.03	0.86 ± 0.03	0.85 ± 0.03
Excellent Feature Rate (%)*	78.5	76.2	74.8	71.3	68.5	65.2

*Notes: (1) “Excellent Feature Rate” refers to the proportion of features with ICC > 0.9 (1084 features per modality); (2) Comparison of overall ICC across modalities: No significant differences were observed between NCCT and CE-T1WI (*p* = 0.21) or ADC (*p* = 0.18), with only a slight difference between NCCT and T1WI (*p* = 0.04); (3) Data were derived from two independent segmentation rounds by two radiologists

### Diagnostic efficacy of models in CE regions

**Fig. 3:**
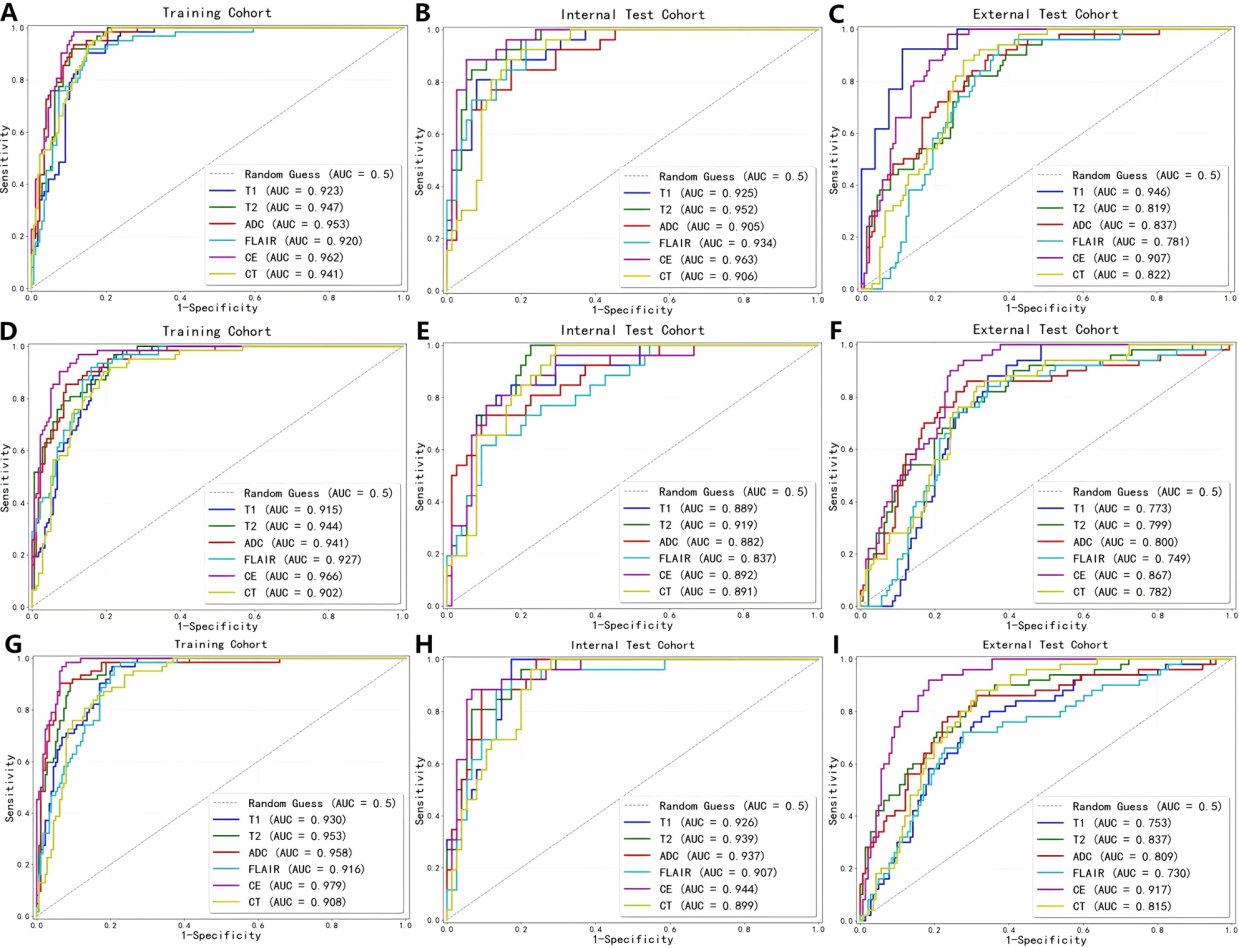
ROC curves for five MRI sequences (T2WI, T1WI, ADC, FLAIR, CE-T1WI) and CT model in the training **(A)**, internal test **(B)**, and external test **(C)** cohorts of the CE region; ROC curves for five MRI sequences and CT models in the training **(D)**, internal test **(E)**, and external test **(F)** cohorts of the NE region; ROC curves for five MRI sequences and CT models in the training **(G)**, internal test **(H)**, and external test **(I)** cohorts of the total tumor region. AUC, area under the receiver operator characteristic curve; ROC, receiver operating characteristic; T2WI, T2-weighted imaging; T1WI, T1-weighted imaging; ADC, apparent diffusion coefficient; FLAIR, fluid-attenuated inversion recovery; CE-T1WI, contrast-enhanced T1WI

As show in Table 3, the CE-T1WI model achieved the highest AUC, which was 0.962 (95% CI: 0.941, 0.984), 0.963 (95% CI: 0.929, 0.996), and 0.907 (95%CI: 0.867, 0.945) in the training, internal test, and external test cohorts, respectively. This was followed by the ADC model, with AUCs of 0.953 (95% CI: 0.928, 0.978), 0.905 (95% CI: 0.843, 0.967), and 0.837 (95%CI: 0.773, 0.896) in the training, internal test, and external test cohorts, respectively. The CT-based model showed AUCs of 0.941 (95% CI: 0.914, 0.968), 0.906 (95% CI: 0.849, 0.963), and 0.822 (95%CI: 0.765, 0.877). Statistical comparisons via the DeLong test revealed a significant difference between the CE-T1WI and CT models (ΔAUC = 0.021, *p* = 0.02), while no significant differences were observed between CT and other MRI sequences (T2WI: ΔAUC = 0.006, *p* = 0.59; T1WI: ΔAUC = 0.022, *p* = 0.72; ADC: ΔAUC = 0.012, *p* = 0.21; FLAIR: ΔAUC = 0.021, *p* = 0.73)(Fig. 3A B C and Table 4). After ComBat harmonization, all modality-specific models exhibited a significant improvement in performance in the external validation cohort. The detailed performance metrics of each modality before and after harmonization, as well as the magnitude of AUC improvement, have been compiled in Additional file 3.

**Table 3:** The best-performing models in each sequence in terms of performance in the CE regions, the NE regions and total tumor regions

Sequence (region)	Cohort	AUC	95%CI	ACC	SEN	SPE
T2WI (CE)	Training Internal test External test	0.947 0.952 0.819	(0.921, 0.973) (0.914, 0.990) (0.757–0.880)	0.899 0.709 0.738	0.919 0.946 0.820	0.892 0.632 0.709
T1WI (CE)	Training Internal test External test	0.923 0.925 0.946	(0.890, 0.955) (0.874, 0.977) (0.871–0.995)	0.866 0.891 0.875	0.903 0.808 0.846	0.852 0.920 0.890
ADC (CE)	Training Internal test External test	0.953 0.905 0.837	(0.928, 0.978) (0.843, 0.967) (0.773, 0.896)	0.899 0.832 0.716	0.936 0.846 0.880	0.886 0.827 0.657
FLAIR (CE)	Training Internal test External test	0.920 0.934 0.781	(0.885, 0.956) (0.889, 0.979) (0.712, 0.843)	0.832 0.822 0.711	0.936 0.760 0.940	0.796 0.591 0.629
CE-T1WI (CE)	Training Internal test External test	0.962 0.963 0.907	(0.941, 0.984) (0.929, 0.996) (0.867, 0.945)	0.912 0.931 0.806	0.984 0.885 0.920	0.886 0.947 0.767
CT (CE)	Training Internal test External test	0.941 0.906 0.822	(0.914, 0.968) (0.849, 0.963) (0.765, 0.877)	0.866 0.852 0.759	0.962 0.885 0.860	0.835 0.840 0.723
T2WI (NE)	Training Internal test External test	0.944 0.919 0.799	(0.917, 0.971) (0.867, 0.971) (0.728–0.864)	0.836 0.832 0.670	0.952 1.000 0.900	0.796 0.773 0.589
T1WI (NE)	Training Internal test External test	0.915 0.889 0.773	(0.881, 0.949) (0.820, 0.959) (0.701,0.835)	0.823 0.852 0.711	0.968 0.808 0.860	0.773 0.867 0.657
ADC (NE)	Training Internal test External test	0.941 0.882 0.800	(0.912, 0.970) (0.809, 0.955) (0.720,0.875)	0.895 0.852 0.753	0.855 0.731 0.860	0.909 0.893 0.714
FLAIR (NE)	Training Internal test External test	0.927 0.837 0.749	(0.896, 0.959) (0.753, 0.921) (0.672,0.824)	0.861 0.832 0.707	0.919 0.615 0.840	0.841 0.907 0.659
CE-T1WI (NE)	Training Internal test External test	0.966 0.892 0.867	(0.945, 0.986) (0.822, 0.961) (0.813,0.910)	0.899 0.772 0.775	0.968 0.962 0.920	0.875 0.707 0.723
CT (NE)	Training Internal test External test	0.902 0.891 0.782	(0.863, 0.942) (0.827, 0.951) (0.710,0.846)	0.832 0.792 0.728	0.903 1.000 0.840	0.807 0.720 0.688
T2WI (Total)	Training Internal test External test	0.953 0.939 0.837	(0.928, 0.978) (0.895, 0.983) (0.774, 0.895)	0.908 0.842 0.728	0.919 0.962 0.840	0.903 0.800 0.688
T1WI (total)	Training Internal test External test	0.930 0.926 0.753	(0.899, 0.960) (0.877, 0.974) (0.672.0.829)	0.840 0.871 0.712	0.968 1.000 0.760	0.796 0.827 0.695
ADC (total)	Training Internal test External test	0.958 0.937 0.809	(0.930, 0.986) (0.892, 0.982) (0.731,0.883)	0.929 0.901 0.726	0.903 0.885 0.840	0.938 0.907 0.686
FLAIR (total)	Training Internal test External test	0.916 0.907 0.730	(0.882, 0.950) (0.844, 0.969) (0.654,0.816)	0.836 0.871 0.723	0.968 0.885 0.720	0.790 0.867 0.723
CE-T1WI (total)	Training Internal test External test	0.979 0.944 0.917	(0.965, 0.993) (0.898, 0.990) (0.877, 0.955)	0.937 0.921 0.843	0.984 0.885 0.920	0.921 0.933 0.816
CT (total)	Training Internal test External test	0.908 0.899 0.815	(0.872, 0.945) (0.841–0.957) (0.752,0.872)	0.807 0.822 0.733	0.936 0.962 0.860	0.761 0.773 0.688

T2WI, T2-weighted imaging; T1WI, T1-weighted imaging; FLAIR, fluid-attenuated inversion recovery; ADC, apparent diffusion coefficient; CE-T1WI, contrast-enhanced T1WI; AUC, area under the curve; CI, confidence interval; ACC, accuracy; SEN, sensibility; SPE, specific

**Table 4:** Results of DeLong’s test of the best models between different sequence MRI models and CT models in the CE regions, NE regions and total tumor regions

	Difference between areas	z statistic	*p*-Value^a^	*p*-Value^b^	*p*-Value^c^
CT vs. T2	0.014	0.536	0.59	-	-
CT vs. T1	0.009	0.365	0.72	-	-
CT vs. ADC	0.033	1.251	0.21	-	-
CT vs. FLAIR	0.0086	0.341	0.73	-	-
CT vs. CE-T1WI	0.0544	2.360	**0.018**	-	-
CT vs. T2	0.042	1.434	-	0.15	-
CT vs. T1	0.021	0.667	-	0.51	-
CT vs. ADC	0.033	1.122	-	0.26	-
CT vs. FLAIR	0.032	1.124	-	0.26	-
CT vs. CE-T1WI	0.081	3.083	-	**0.002**	-
CT vs. T2	0.001	0.058	-	-	0.95
CT vs. T1	0.038	1.283	-	-	0.20
CT vs. ADC	0.035	1.394	-	-	0.16
CT vs. FLAIR	0.016	0.544	-	-	0.59
CT vs. CE-T1WI	0.062	2.821	-	-	**0.005**

Bold type indicate *p* < 0.05

*p*-Value^a^ is for Comparison of CT models in the CE regions with five MRI sequences

*p*-Value^b^ is for Comparison of CT models in NE regions with five MRI sequences

*p*-Value^c^ is for Comparison of CT models in total tumor regions with five MRI sequences

T2WI, T2-weighted imaging; T1WI, T1-weighted imaging; FLAIR, fluid-attenuated inversion recovery; CE-T1WI, contrast-enhanced T1WI; ADC, apparent diffusion coefficient

### Diagnostic efficacy of models in NE regions

For the NE region, the CE-T1WI model maintained superior performance with an AUC of 0.966 (95% CI: 0.945, 0.986), 0.892 (95% CI: 0.822, 0.961), and 0.867 (95% CI: 0.813,0.910) in the training, internal test, and external test cohorts, respectively (Table 3). Significant differences were observed between CE-T1WI and CT models (ΔAUC = 0.064, *p* = 0.002). In contrast, CT performance was statistically comparable to T2WI (ΔAUC = 0.042, *p* = 0.15), T1WI (ΔAUC = 0.013, *p* = 0.51), ADC (ΔAUC = 0.039, *p* = 0.26) and FLAIR (ΔAUC = 0.025, *p* = 0.26)(Fig. 3D E F and Table 4).

### Diagnostic efficacy of combined models combining CE and NE regions

**Fig. 4:**
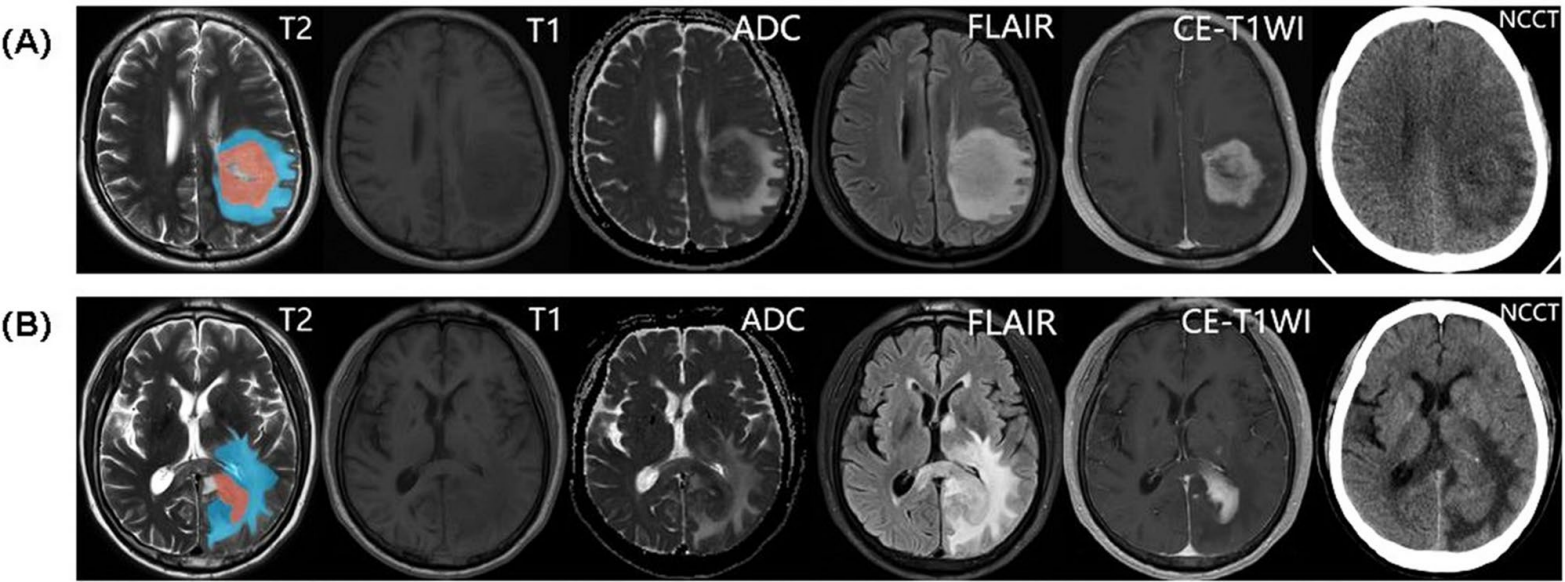
Multimodal imaging findings of representative cases of GBM (**A**) and PCNSL (**B**). From left to right: T2-weighted imaging (T2WI), T1-weighted imaging (T1WI), apparent diffusion coefficient (ADC) map, fluid-attenuated inversion recovery (FLAIR), contrast-enhanced T1-weighted imaging (CE-T1WI), and non-contrast computed tomography (NCCT). ROI masks are overlaid on the leftmost T2WI: CE region (red), and NE region (blue)

When combining features from both CE and NE regions, the CE-T1WI model achieved the highest AUC, which was 0.979 (95% CI: 0.965, 0.993), 0.944 (95% CI: 0.898, 0.990), and 0.917 (95% CI: 0.877, 0.955) in the training, internal test, and external test cohorts respectively, whereas the combined CT model had an AUC of 0.908 (95% CI: 0.872, 0.945), 0.899 (95% CI: 0.841, 0.957), and 0.815 (95% CI: 0.752, 0.872)(Table 3). The CE-T1WI model significantly outperformed the CT model (ΔAUC = 0.071, *p* = 0.005) (Table 3). No significant differences were observed between CT and other MRI sequences in the whole tumor region (T2WI: ΔAUC = 0.045, *p* = 0.95; T1WI: ΔAUC = 0.022, *p* = 0.20; ADC: ΔAUC = 0.050, *p* = 0.16; FLAIR: ΔAUC = 0.008, *p* = 0.59)(Fig. 3G H I and Table 4). The representative cases with GBM and PCNSL are given in Fig. 4.

### Subgroup analysis of model performance by device vendor

To further verify the robustness of models across different device vendors (a key concern for clinical translation), we stratified the external test cohort (204 cases from Center2 and Center3) by scanner vendor and re-evaluated diagnostic efficacy. Specifically, Center2 used Philips devices (MRI: Ingenia CX; CT: iCT V4.17) and Center3 used GE devices (MRI: Signa HDxt; CT: Revolution Apex). Stratified results showed that for all modalities, the AUC fluctuation across vendors was ≤ 5.2%, and all AUCs remained within the clinically effective range (AUC > 0.8): For the CE-T1WI model (the optimal modality), AUCs were 0.896 (Philips) and 0.889 (GE) in the CE region; for the CT model, AUCs were 0.815 (Philips) and 0.807 (GE) in the CE region. These findings confirm that the model’s performance is not significantly affected by scanner vendor differences. Detailed stratified metrics are provided in Additional file 4.

### Model explanation

**Fig. 5:**
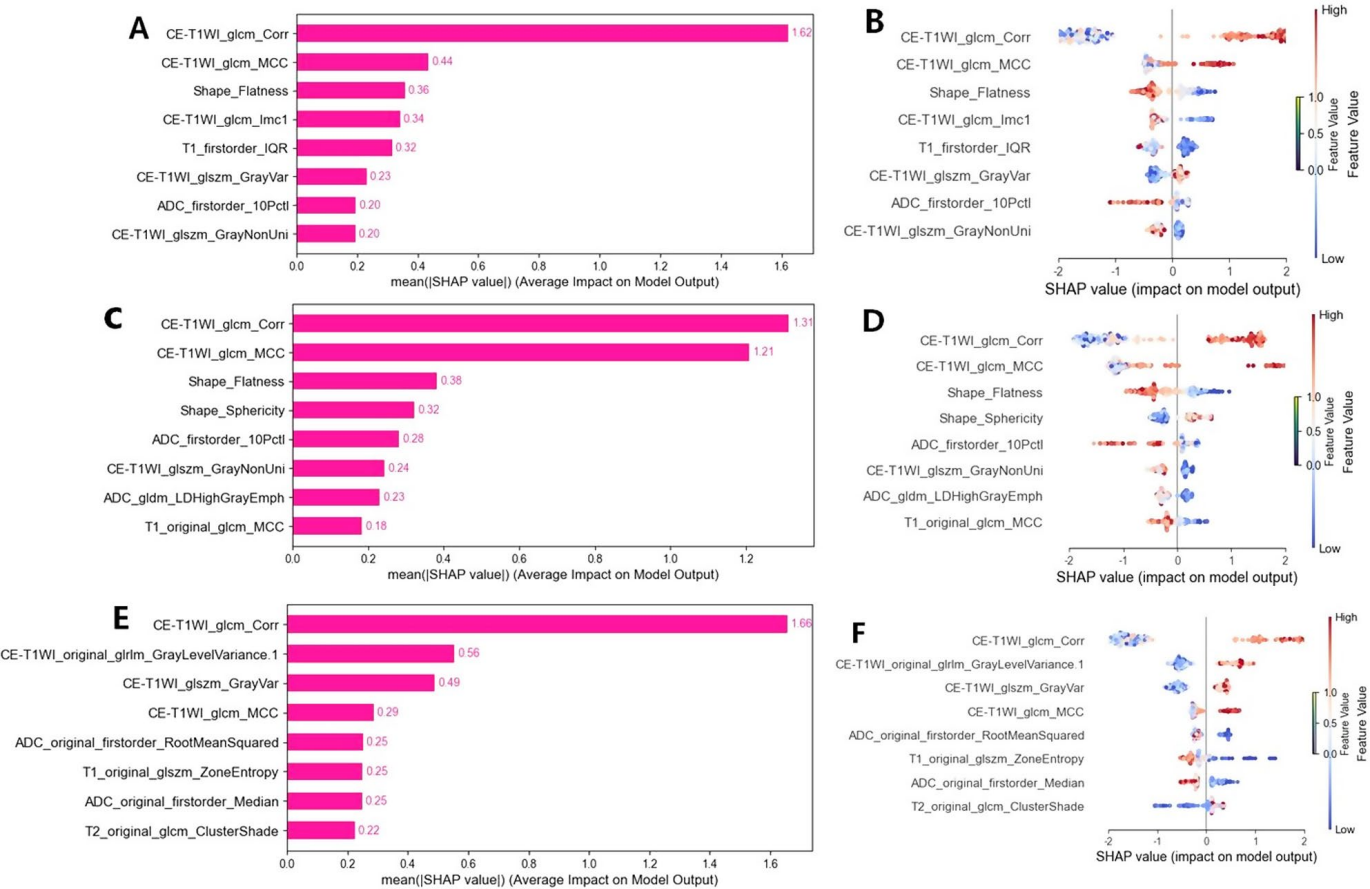
SHAP analysis of radiomic feature contributions. (**A**,** C**,** E**) Bar charts showing which radiomic features most strongly influence model predictions for the CE region, NE region, and combined tumor models. CE-T1WI_glcm_Correlation stands out as the most influential feature across all models. (**B**,** D**,** F**) SHAP dependence plots showing how feature values affect model outcomes: positive values favor a GBM diagnosis, while negative values favor PCNSL. Red points represent high feature values, and blue points represent low values

Figure 5 presents the feature contributions of the CE region, NE region, and combined models via SHAP analysis, shown as feature importance rankings (mean absolute SHAP values) and dependence plots. For the CE region model, CE-T1WI_glcm_Correlation (mean |SHAP| = 1.62) was the most impactful feature, followed by CE-T1WI_glcm_MCC (0.44) and Shape_Flatness (0.36); for the NE region model, CE-T1WI_glcm_Correlation (mean |SHAP| = 1.31) remained the primary driver, followed by CE-T1WI_glcm_MCC (1.21), notably with morphological (Shape_Sphericity, 0.32) and diffusion (ADC_firstorder_10Pctl, 0.28) features also contributing; for the combined model, CE-T1WI_glcm_Correlation (mean |SHAP| = 1.66) again dominated, followed by CE-T1WI_original_glcm_GrayLevelVariance (0.56) and CE-T1WI_glszm_GrayVariance (0.49), with T1/T2 sequence features (e.g., T1_original_glszm_ZoneEntropy, 0.25; T2_original_glcm_ClusterShade, 0.22) adding complementary value. Across all models, CE-T1WI_glcm_Correlation consistently served as the core predictive feature, highlighting the critical role of CE-region texture uniformity in differentiating GBM from PCNSL, while region-specific supplementary features enhanced model robustness and biological interpretability by linking radiomic features to tumor cellularity, necrosis, and tissue heterogeneity.

## Discussion

This study systematically evaluated the efficacy of radiomic models based on NCCT and multiparametric MRI in the differential diagnosis of GBM and PCNSL. The results showed that the CE-T1WI-based radiomics model demonstrated the best diagnostic performance. Of particular importance, the diagnostic efficacy of the NCCT model in both CE and NE regions was not statistically significantly different from that of any other MRI sequences (T2WI, T1WI, ADC, and FLAIR) except for the CE-T1WI model (DeLong test, *p* > 0.05). This finding strongly supports the NCCT radiomics model as a viable alternative for the differential diagnosis of GBM and PCNSL in scenarios where MRI resources are limited or contraindications exist.

CE-T1WI radiomics has shown excellent diagnostic efficacy in both CE and NE regions and its advantage stems from its ability to capture key tumor biological characteristics that are distinct and complementary between the two regions. SHAP analysis further confirms that the core driving feature of the CE-T1WI model is CE-T1WI_glcm_Correlation, which quantifies the texture uniformity of the enhancing region to directly link differences in tumor angiogenesis and cell density, making it a key discriminative marker across CE, NE, and combined models. In the CE region, the model accurately quantified differences in tumor angiogenesis, blood-brain barrier disruption, and necrosis patterns [16, 17]. The heterogeneous enhancement of GBM is closely associated with its hypervascularity, disruption of the blood-brain barrier (BBB), and pseudopalisading necrosis, characterized by irregular accumulation of contrast agent at the margins of necrotic foci [18, 19]. This pathological feature leads to homogeneous grayscale distribution on CE-T1WI, corresponding to higher CE-T1WI_glcm_Correlation values. In contrast, the homogeneous enhancement pattern of PCNSL reflects dense lymphoid cell infiltration and a relatively intact BBB [20]. Shim et al. [21] further validated this regional specificity: they found that the CE region of GBM primarily encodes biological information related to blood vessels and the BBB, which is completely non-overlapping with the information carried by the NE region. In the NE region, GBM is not purely vasogenic edema but contains a large number of infiltrating tumor cells. Eidel O et al. demonstrated that the NE region of GBM actually contains a high density of infiltrating tumor cells, and that its active cell density can be comparable to that of the CE region [22]. Building on this, Shim et al. [21] supplemented that the NE region also harbors critical information on tumor cell infiltration and microenvironmental heterogeneity—features particularly important for predicting local recurrence of GBM. This dense cell infiltration leads to irregular margins of the NE region, corresponding to lower Shape_Sphericity values. In contrast, the NE region of PCNSL was dominated by simple edema with a lower degree of tumor cell infiltration, resulting in more regular necrosis morphology and higher Shape_Sphericity values [16]. This microscopic difference was quantified by radiomic characterization, which compensated for the limitations of conventional MRI morphology analysis.

The results of this study further validate the diagnostic value of other MRI sequence models. The ADC-based model effectively reflects the core difference between the significant diffusion limitation due to the high nuclear-to-mass ratio of PCNSL and the relatively free diffusion of the necrotic zone of GBM by quantifying the diffusion properties of water molecules [7, 23]. In SHAP analysis, ADC_firstorder_10Pctl served as a key supplementary feature in the NE region model: lower values indicate restricted diffusion and support PCNSL diagnosis via positive SHAP values.T2WI and FLAIR models provide additional information by reflecting differences in peritumoral edema and tissue water content, emphasizing that the peritumoral region contains critical biological information that can be effectively mined by radiomics [24–27]. Notably, the relatively low validity of the T1WI model is related to its lack of ability to characterize tumor vascular details. This highlights the limitations of single-sequence morphological analysis and emphasizes the advantages of combined analysis with multiparameter MRI (e.g., CE-T1WI combined with ADC) [10].

A notable finding of this study is the diagnostic efficacy of the CT radiomics model in both the CE and NE regions as equivalent to MRI sequences other than CE-T1WI. Among them, CT and ADC have the most similar efficacy, suggesting that they may quantify the core pathological feature of “cell density” in different dimensions: ADC directly reflects the extent to which the diffusion of water molecules is limited by the high nucleoplasmic ratio (at the level of microscopic molecular motion), whereas NCCT indirectly characterizes the degree of cellular densification (macroscopic density texture level) through the heterogeneity of the distribution of tissue attenuation (HU) [14, 28]. T2 and FLAIR directly show peritumoral edema through the relaxation properties of water molecules, T1WI relies on tissue T1 relaxation time differences to show anatomical structures, and NCCT also effectively reflects tumor heterogeneity through density texture [19, 29, 30].

The introduction of an external validation cohort (from an independent center) to assess model generalization capability is a key strength of this study, and its results show a slight decrease in the AUC of each model compared to the internal validation, reflecting potential differences in scanning parameters, image post-processing, and highlighting the importance of the model’s generalization capability between different institutions. It is worth emphasizing that the NCCT model showed superior stability under external validation conditions: its AUC in the CE region decreased by only 8.1%, which was significantly lower than that of the MRI model (e.g., 19.7% for the T1WI model). This may be attributed to the superior cross-device / platform reproducibility of CT, which makes its radiomics profile relatively unaffected by center-to-center variation. Our supplementary ICC analysis further confirmed this advantage: the overall mean ICC of NCCT radiomic features was 0.90 ± 0.02, comparable to non-contrast MRI sequences (T1WI: 0.85 ± 0.03, T2WI: 0.87 ± 0.03), with slightly higher ICC in morphological (0.93 ± 0.01) and first-order histogram features (0.91 ± 0.01), providing quantitative evidence for its stable cross-center performance.

This study has several limitations. First, as a retrospective study, it included data from three clinical centers for external validation but had a relatively limited sample size (total cases < 600), which may restrict the model’s generalization in clinical settings with different geographic regions and imaging devices. Future multicenter prospective studies are needed to further validate the model’s stability. Second, radiomic feature extraction relies on accurate tumor segmentation. Although we adopted semi-automatic segmentation combined with expert refinement to balance efficiency and accuracy, a certain degree of subjectivity remained in the segmentation process. Subsequent research can incorporate multimodal image fusion segmentation technology to further reduce artificial delineation errors. Furthermore, only traditional machine learning models were used to construct the diagnostic framework in the initial analysis, with no deep learning or hybrid radiomics-deep learning models included, which limited the exploration of algorithmic performance boundaries. To address this limitation, we additionally built a ResNet50 deep learning model for benchmark comparison, using the identical training/test cohorts and imaging input (CE-T1WI enhanced region images, the optimal diagnostic modality in this study) as the traditional models. Results revealed no significant difference in their generalization performance: the optimal traditional model and ResNet50 achieved AUCs of 0.963 and 0.958 in the internal test set, and 0.907 and 0.892 in the external test set, respectively. This finding aligns with recent studies that, with a moderate sample size (*n* < 600) and rigorous radiomic feature quality control, traditional machine learning models deliver performance comparable to deep learning models due to the stability of their structured features [31, 32]. Moreover, traditional models enable feature importance visualization via SHAP analysis, conferring an irreplaceable interpretability advantage that helps clinicians understand the diagnostic rationale and facilitates the model’s clinical translation.

In conclusion, while CE-T1WI exhibit highest diagnostic efficacy in differentiating GBM from PCNSL, NCCT achieves comparable diagnostic efficacy to most MRI sequences and demonstrates superior cross-center stability. This establishes NCCT as a practical alternative for patients with MRI contraindications or in resource-constrained settings, enhancing precision in treatment planning.

## Supplementary Information

Below is the link to the electronic supplementary material.

Supplementary Material 1: File name: Additional file 1. File format: .pdf. Title of data: Detailed MR and CT Imaging Protocols. Description of data: Presents standardized imaging protocols for 3 study centers, including 3.0T MRI and multi-detector spiral CT scanners. Includes center-specific parameter adjustments (validated to not affect quality), scanner models/sequence parameters (Tables S1-1 for MRI, S1-2 for CT), and notes (all CT scans are NCCT; abbreviations like CE-T1WI, DWI defined).

Supplementary Material 2: File name: Additional file 2. File format: .pdf. Title of data: Supplementary Data on Radiomic Feature Screening, Stability Verification, and Preprocessing Effects. Description of data: Supports feature selection/model reproducibility with 3 tables: S1 (IBSI v2-compliant selected features, SHAP values, 5-fold frequency); S2 (stable features’ 5-fold validation metrics like ICC); S3 (Z-score normalized features of 30 cases, GBM/PCNSL ratio consistent with total sample).

Supplementary Material 3: File name: Additional file 3. File format: .pdf. Title of data: AUC Values of Radiomics Models Before and After ComBat Correction. Description of data: Compares AUC (with 95% CI) of radiomics models for 6 sequences (CE-T1WI, CT, etc.) and 3 tumor regions (CE, NE, Total) in uncorrected (including Center3) and ComBat-corrected scenarios; correction improves AUC in most cases (e.g., CE-T1WI Total: 0.875→0.917).

Supplementary Material 4: File name: Additional file 4. File format: .pdf. Title of data: External Validation Performance of Radiomics Models Stratified by Device Vendor. Description of data: Shows external validation metrics (AUC, ACC, SEN, SPE) of ComBat-harmonized models (Philips/GE vendors). Validation cohort: Center2 (Philips, n = 103), Center3 (GE, n = 101); CE-T1WI/T1WI perform well, metrics consistent across vendors (abbreviations: GBM, PCNSL, CE, NE defined).

## Data Availability

The datasets used and/or analysed during the current study are available from the corresponding author on reasonable request.

## Abbreviations

GBM: Glioblastoma
PCNSL: Primary central nervous system lymphoma
NCCT: Noncontrast CT
T2WI: T2-weighted imaging
T1WI: T1-weighted imaging
ADC: Apparent diffusion coefficient
FLAIR: Fluid-attenuated inversion recovery
CE-T1WI: Contrast-enhanced T1WI
CE: Contrast-enhancing
NE: Non-enhancing
DWI: Diffusion-weighted imaging
DSC-PWI: Dynamic sensitive contrast-enhanced perfusion-weighted imaging
ITSS: Intratumoral susceptibility signals intensity
SWI: Susceptibility-weighted imaging
ICC: Intragroup correlation coefficient
RFE: Recursive feature elimination
SVM: Support vector machine
AUC: Area under curve
LDA: Linear discriminant Analysis
LR: Logistic regression
DT: Decision tree
ACC: Accuracy
SEN: Sensitivity
SPE: Specificity
BBB: Blood-brain barrier
