# Supplementary material for: Radiomics-based differentiation between glioblastoma and primary central nervous system lymphoma: CT vs MRI
Source: Cancer Imaging. 2026 Mar 16;26:54. doi: 10.1186/s40644-026-01018-8 (PMC13104201; doi:10.1186/s40644-026-01018-8)
Supplement: Supplementary file 1 — Supplementary Material 1: File name: Additional file 1. File format: .pdf. Title of data: Detailed MR and CT Imaging Protocols. Description of data: Presents standardized imaging protocols for 3 study centers, including 3.0T MRI and multi-detector spiral CT scanners. Includes center-specific parameter adjustments (validated to not affect quality), scanner models/sequence parameters (Tables S1-1 for MRI, S1-2 for CT), and notes (all CT scans are NCCT; abbreviations like CE-T1WI, DWI defined). [file 40644_2026_1018_MOESM1_ESM.pdf]

Detailed MR and CT Imaging Protocols

1. General Equipment and Sequence Description

All three centers involved in this study implemented standardized imaging protocols. MRI examinations were uniformly performed using 3.0 Tesla (T) scanners, while CT examinations employed multi-detector spiral CT scanners. Center-specific adjustments were made to core parameters to accommodate differences in hardware characteristics across devices. Detailed specifications of scanner models, sequence parameters, and scanning modes are summarized in Tables S1-1 (MRI) and S1-2 (CT) below. All devices operated in standard spiral mode to minimize inter-device variability in image reconstruction parameters.

2. MRI Imaging Protocol

Parameter	Center 1 (Siemens)	Center 2 (Philips)	Center 3 (GE)
Scanner Model	MAGNETOM Prisma/Skyra	3.0T PHILIPS Ingenia CX	Signa HDxt 3.0Tesla
Core Sequences	T2WI, T1WI, FLAIR, CE-T1WI, DWI	T2WI, T1WI, FLAIR, CE-T1WI, DWI	T2WI, T1WI, FLAIR, CE-T1WI, DWI
Field of View (FOV)	220×220 mm^2	220×220 mm^2	220×220 mm^2
Slice Thickness	5 mm	5 mm	6 mm (adjusted for device compatibility)
Matrix	256×256	256×256	320×320
Slice Gap	1 mm	1 mm	1 mm
DWI Parameters	Spin-echo echo-planar imaging sequence; b-values = 0/1000 s/mm^2; 3 orthogonal diffusion-sensitive gradients	Same as left	Same as left
ADC Map Generation	Automatically generated by MRI workstation	Automatically generated by MRI workstation	Automatically generated by MRI workstation

3. CT Imaging Protocol

Parameter	Center 1 (Canon/Philips)	Center 2 (Philips)	Center 3 (GE)
Scanner Model	Aquilion ONE (320-slice)/Brilliance iCT (256-slice)	256-slice PHILIPS iCT V4.17	Revolution Apex 256-detector
Slice Thickness	5 mm	5 mm	5 mm
Tube Current	200-300 mA	350 mA	390 mA
Tube Voltage	120 kV	120 kV	120 kV
Matrix	512×512	512×512	512×512
Field of View (FOV)	250 mm	250 mm	500 mm (adjusted for device compatibility)
Scanning Mode	Standard spiral mode	Standard spiral mode	Standard spiral mode

Notes： Center-specific parameter adjustments (e.g., slice thickness, FOV in Center 3) were validated to ensure no impact on image quality or quantitative analysis;All CT scans were non-contrast enhanced (NCCT) ;CE-T1WI: Contrast-enhanced T1-weighted imaging; DWI: Diffusion-weighted imaging; ADC: Apparent diffusion coefficient.
