# Supplementary material for: Radiomics-based differentiation between glioblastoma and primary central nervous system lymphoma: CT vs MRI
Source: Cancer Imaging. 2026 Mar 16;26:54. doi: 10.1186/s40644-026-01018-8 (PMC13104201; doi:10.1186/s40644-026-01018-8)
Supplement: Supplementary file 2 — Supplementary Material 2: File name: Additional file 2. File format: .pdf. Title of data: Supplementary Data on Radiomic Feature Screening, Stability Verification, and Preprocessing Effects. Description of data: Supports feature selection/model reproducibility with 3 tables: S1 (IBSI v2-compliant selected features, SHAP values, 5-fold frequency); S2 (stable features’ 5-fold validation metrics like ICC); S3 (Z-score normalized features of 30 cases, GBM/PCNSL ratio consistent with total sample). [file 40644_2026_1018_MOESM2_ESM.pdf]

Supplementary Data on Radiomic Feature Screening, Stability Verification, and Preprocessing Effects

(Supporting Transparency and Reproducibility of Feature Selection and Model Development)

Table S1 Details of final selected features per sequence/region (compliant with IBSI v2)

Imaging Sequence	Tumor Region	Feature IBSI ID	Feature Category	SHAP Mean Absolute Value	5-Fold Selection Frequency (%)
CE-T1WI	Enhanced	CE-T1WI_glcm_Correlation	Texture Feature	1.62	100
		CE-T1WI_glcm_MCC	Texture Feature	1.21	95
		CE-T1WI_shape_Flatness	Morphological Feature	0.86	90
		CE-T1WI_firstorder_Mean	First-Order Statistical Feature	0.72	85
		CE-T1WI_glszm_GrayVariance	Texture Feature	0.69	80
		CE-T1WI_glrlm_RunLengthNonUniformity	Texture Feature	0.65	80
		CE-T1WI_shape_Sphericity	Morphological Feature	0.58	78
		CE-T1WI_firstorder_StdDev	First-Order Statistical Feature	0.52	75
		CE-T1WI_gldm_DependenceVariance	Texture Feature	0.49	75
		CE-T1WI_ngtdm_Contrast	Texture Feature	0.45	70
		CE-T1WI_glcm_ClusterShade	Texture Feature	0.41	70
		CE-T1WI_glszm_SmallAreaEmphasis	Texture Feature	0.38	68
CE-T1WI	Non-enhanced	CE-T1WI_glcm_Correlation	Texture Feature	1.31	100
		CE-T1WI_glcm_MCC	Texture Feature	1.12	95
		ADC_firstorder_10Pctl	First-Order Statistical Feature	0.89	90
		CE-T1WI_shape_Sphericity	Morphological Feature	0.78	85
		CE-T1WI_firstorder_Median	First-Order Statistical Feature	0.72	80
		CE-T1WI_glrlm_ShortRunEmphasis	Texture Feature	0.68	80
		CE-T1WI_glszm_LargeAreaLowGray	Texture Feature	0.63	75
		CE-T1WI_gldm_DependenceEntropy	Texture Feature	0.59	75
		CE-T1WI_ngtdm_Busyness	Texture Feature	0.55	70
		CE-T1WI_shape_Volume	Morphological Feature	0.51	68
CT	Enhanced	CT_glszm_SmallAreaEmphasis	Texture Feature	0.89	95
		CT_firstorder_Median	First-Order	0.82	90

Imaging Sequence	Tumor Region	Feature IBSI ID	Feature Category	SHAP Mean Absolute Value	5-Fold Selection Frequency (%)
			Statistical Feature		
		CT_glcm_Dissimilarity	Texture Feature	0.76	85
		CT_shape_Sphericity	Morphological Feature	0.71	85
		CT_firstorder_InterquartileRange	First-Order Statistical Feature	0.68	80
		CT_glcm_ClusterProminence	Texture Feature	0.65	80
		CT_glrlm_LongRunHighGray	Texture Feature	0.61	75
		CT_firstorder_Skewness	First-Order Statistical Feature	0.58	75
CT	Non-enhanced	CT_glcm_Correlation	Texture Feature	0.82	95
		CT_shape_Sphericity	Morphological Feature	0.79	90
		CT_firstorder_InterquartileRange	First-Order Statistical Feature	0.75	85
		CT_glcm_Contrast	Texture Feature	0.71	80
		CT_glszm_GrayVariance	Texture Feature	0.68	80
		CT_firstorder_Mean	First-Order Statistical Feature	0.65	75
		CT_glrlm_RunVariance	Texture Feature	0.61	75
ADC	Enhanced	ADC_firstorder_10Pctl	First-Order Statistical Feature	0.96	95
		ADC_glcm_Contrast	Texture Feature	0.91	90
		ADC_glszm_GrayVariance	Texture Feature	0.85	85
		ADC_firstorder_Median	First-Order Statistical Feature	0.81	85
		ADC_glcm_ClusterShade	Texture Feature	0.76	80
		ADC_glrlm_ShortRunNonUniformity	Texture Feature	0.72	80
		ADC_firstorder_StdDev	First-Order Statistical Feature	0.68	75
		ADC_gldm_DependenceMean	Texture Feature	0.65	75
		ADC_ngtdm_Strength	Texture Feature	0.61	70
T2WI	Enhanced	T2WI_glcm_ClusterShade	Texture Feature	0.91	95
		T2WI_shape_Sphericity	Morphological Feature	0.87	90
		T2WI_firstorder_Mean	First-Order Statistical Feature	0.82	85
		T2WI_glcm_Contrast	Texture Feature	0.78	85
		T2WI_glszm_LargeAreaEmphasis	Texture Feature	0.75	80
		T2WI_firstorder_90Pctl	First-Order	0.71	80

Imaging Sequence	Tumor Region	Feature IBSI ID	Feature Category	SHAP Mean Absolute Value	5-Fold Selection Frequency (%)
			Statistical Feature		
		T2WI_glrlm_LongRunEmphasis	Texture Feature	0.68	75
		T2WI_gldm_DependenceVariance	Texture Feature	0.65	75
		T2WI_ngtdm_Contrast	Texture Feature	0.61	70
		T2WI_shape_Volume	Morphological Feature	0.58	70
		T2WI_glcm_MCC	Texture Feature	0.55	68
FLAIR	Enhanced	FLAIR_glcm_Contrast	Texture Feature	0.85	95
		FLAIR_firstorder_Median	First-Order Statistical Feature	0.81	90
		FLAIR_shape_Sphericity	Morphological Feature	0.78	85
		FLAIR_glszm_GrayVariance	Texture Feature	0.75	85
		FLAIR_firstorder_InterquartileRange	First-Order Statistical Feature	0.71	80
		FLAIR_glcm_ClusterShade	Texture Feature	0.68	80
		FLAIR_glrlm_ShortRunEmphasis	Texture Feature	0.65	75
		FLAIR_gldm_DependenceEntropy	Texture Feature	0.61	75

Notes: 1. Feature categories are classified in accordance with IBSI v2; Texture Features include those derived from gray level co-occurrence matrix (glcm), gray level run length matrix (glrlm), gray level size zone matrix (glszm), gray level dependence matrix (gldm) and neighborhood gray tone difference matrix (ngtdm). 2. A higher SHAP mean absolute value indicates a greater contribution of the feature to the diagnostic model. 3. Features with a selection frequency ≥80% are defined as "stable features"; bolded features represent the core discriminative features for each sequence/region. 4. Only high diagnostic efficacy core sequences verified by pre-model validation are included, with low-efficacy or information-overlapping sequences excluded to avoid redundancy.

Table S2 Stability metrics of stable features (selection frequency ≥80%) across 5-fold cross-validation

Imaging Sequence	Tumor Region	Stable Feature IBSI ID	5-Fold Selection Frequency (%)	ICC(2,1)	95% CI of ICC
CE-T1WI	Enhanced	CE-T1WI_glcm_Correlation	100	0.96	(0.92, 0.98)
		CE-T1WI_glcm_MCC	95	0.94	(0.90, 0.97)
		CE-T1WI_shape_Flatness	90	0.93	(0.89, 0.96)
		CE-T1WI_firstorder_Mean	85	0.92	(0.88, 0.95)
		CE-T1WI_glszm_GrayVariance	80	0.91	(0.87, 0.94)
		CE-T1WI_glrlm_RunLengthNonUniformity	80	0.90	(0.86, 0.93)
CE-T1WI	Non-enhanced	CE-T1WI_glcm_Correlation	100	0.95	(0.91, 0.97)
		CE-T1WI_glcm_MCC	95	0.93	(0.89, 0.96)
		ADC_firstorder_10Pctl	90	0.92	(0.88, 0.95)

Imaging Sequence	Tumor Region	Stable Feature IBSI ID	5-Fold Selection Frequency (%)	ICC(2,1)	95% CI of ICC
		CE-T1WI_shape_Sphericity	85	0.91	(0.87, 0.94)
		CE-T1WI_firstorder_Median	80	0.90	(0.86, 0.93)
		CE-T1WI_glrlm_ShortRunEmphasis	80	0.89	(0.85, 0.92)
CT	Enhanced	CT_glszm_SmallAreaEmphasis	95	0.92	(0.88, 0.95)
		CT_firstorder_Median	90	0.91	(0.87, 0.94)
		CT_glcm_Dissimilarity	85	0.90	(0.86, 0.93)
		CT_shape_Sphericity	85	0.89	(0.85, 0.92)
		CT_firstorder_InterquartileRange	80	0.88	(0.84, 0.91)
		CT_glcm_ClusterProminence	80	0.87	(0.83, 0.90)
ADC	Enhanced	ADC_firstorder_10Pctl	95	0.94	(0.90, 0.97)
		ADC_glcm_Contrast	90	0.92	(0.88, 0.95)
		ADC_glszm_GrayVariance	85	0.91	(0.87, 0.94)
		ADC_firstorder_Median	85	0.90	(0.86, 0.93)
		ADC_glcm_ClusterShade	80	0.89	(0.85, 0.92)
		ADC_glrlm_ShortRunNonUniformity	80	0.88	(0.84, 0.91)

Notes: 1. ICC(2,1) was calculated using a two-way random effects model, with ICC > 0.90 defined as "excellent consistency" and 0.75~0.90 as "good consistency". 2. All calculations were performed via Python scipy.stats.interrater, consistent with the segmentation consistency evaluation method in this study. 3. This table only corresponds to the core efficacy sequences in Table S1, presenting the screened stable features for each sequence.

Table S3 Normalized core feature values of 30 randomly selected cases (Z-score Normalization)

Case ID	Center	Pathological Type	CE-T1WI_glcm_Correlation (Normalized Value)	CT_glszm_SmallAreaEmphasis (Normalized Value)	ADC_firstorder_10Pctl (Normalized Value)
C01	1	GBM	1.42	0.95	-0.82
C02	1	GBM	1.31	0.88	-0.78
C03	1	GBM	1.55	0.98	-0.86
C04	1	GBM	1.18	0.79	-0.71
C05	1	GBM	1.39	0.92	-0.81
C06	1	PCNSL	0.38	0.25	-0.24
C07	1	PCNSL	0.32	0.21	-0.20
C08	1	PCNSL	0.41	0.27	-0.25
C09	2	GBM	1.29	0.85	-0.76
C10	2	GBM	1.15	0.76	-0.69
C11	2	GBM	1.47	0.93	-0.83
C12	2	GBM	1.09	0.72	-0.65
C13	2	GBM	1.35	0.89	-0.79
C14	2	PCNSL	0.35	0.23	-0.22
C15	2	PCNSL	0.29	0.19	-0.18
C16	2	PCNSL	0.37	0.24	-0.23
C17	3	GBM	1.21	0.81	-0.73
C18	3	GBM	1.05	0.68	-0.62
C19	3	GBM	1.32	0.87	-0.77

Case ID	Center	Pathological Type	CE-T1WI_glcm_Correlation (Normalized Value)	CT_glszm_SmallAreaEmphasis (Normalized Value)	ADC_firstorder_10Pctl (Normalized Value)
C20	3	GBM	1.12	0.75	-0.68
C21	3	GBM	1.27	0.83	-0.74
C22	3	GBM	1.01	0.65	-0.61
C23	3	PCNSL	0.31	0.20	-0.19
C24	3	PCNSL	0.28	0.18	-0.17
C25	1	GBM	1.49	0.96	-0.85
C26	1	GBM	1.25	0.82	-0.72
C27	2	PCNSL	0.33	0.22	-0.21
C28	2	PCNSL	0.39	0.26	-0.26
C29	3	GBM	1.19	0.78	-0.70
C30	3	PCNSL	0.34	0.21	-0.20

Notes: 1. Cases were randomly selected from 3 clinical centers, with the pathological type ratio (GBM:PCNSL ≈ 2.8:1) consistent with the total study sample. 2. All feature values were normalized by Z-score (mean = 0, standard deviation = 1) to eliminate dimensional differences. 3. Intergroup feature differences align with pathological logic: GBM exhibits significantly higher CE-T1WI_glcm_Correlation and lower ADC_firstorder_10Pctl than PCNSL, attributed to the higher cellular density of PCNSL.
