# Supplementary material for: Radiomics-based differentiation between glioblastoma and primary central nervous system lymphoma: CT vs MRI
Source: Cancer Imaging. 2026 Mar 16;26:54. doi: 10.1186/s40644-026-01018-8 (PMC13104201; doi:10.1186/s40644-026-01018-8)
Supplement: Supplementary file 3 — Supplementary Material 3: File name: Additional file 3. File format: .pdf. Title of data: AUC Values of Radiomics Models Before and After ComBat Correction. Description of data: Compares AUC (with 95% CI) of radiomics models for 6 sequences (CE-T1WI, CT, etc.) and 3 tumor regions (CE, NE, Total) in uncorrected (including Center3) and ComBat-corrected scenarios; correction improves AUC in most cases (e.g., CE-T1WI Total: 0.875→0.917). [file 40644_2026_1018_MOESM3_ESM.pdf]

AUC values of radiomics models before and after ComBat Correction

	Region	Uncorrected(Including Center3)	Corrected (Including Center3+ComBat)
CE-T1WI	CE	0.886（0.842-0.930）	0.907（0.867-0.945）
	NE	0.823（0.765-0.881）	0.867（0.813-0.910）
	Total	0.875（0.829-0.921）	0.917（0.877-0.955）
CT	CE	0.805（0.742-0.868）	0.822（0.765-0.877）
	NE	0.763（0.697-0.829）	0.782（0.710-0.846）
	Total	0.793（0.728-0.858）	0.815（0.752-0.872）
ADC	CE	0.812（0.746-0.878）	0.837（0.773-0.896）
	NE	0.781（0.712-0.850）	0.800（0.720-0.875）
	Total	0.798（0.731-0.865）	0.809（0.731-0.883）
T2WI	CE	0.795（0.731-0.859）	0.819（0.757-0.880）
	NE	0.778（0.705-0.851）	0.799（0.728-0.864）
	Total	0.813（0.749-0.877）	0.837（0.774-0.895）
T1WI	CE	0.928（0.885-0.971）	0.946（0.871-0.995）
	NE	0.751（0.677-0.825）	0.773（0.701-0.835）
	Total	0.732（0.653-0.811）	0.753（0.672-0.829）
FLAIR	CE	0.763（0.692-0.834）	0.781（0.712-0.843）
	NE	0.728（0.649-0.807）	0.749（0.672-0.824）
	Total	0.712（0.633-0.791）	0.730（0.654-0.816）
