# Supplementary material for: Radiomics-based differentiation between glioblastoma and primary central nervous system lymphoma: CT vs MRI
Source: Cancer Imaging. 2026 Mar 16;26:54. doi: 10.1186/s40644-026-01018-8 (PMC13104201; doi:10.1186/s40644-026-01018-8)
Supplement: Supplementary file 4 — Supplementary Material 4: File name: Additional file 4. File format: .pdf. Title of data: External Validation Performance of Radiomics Models Stratified by Device Vendor. Description of data: Shows external validation metrics (AUC, ACC, SEN, SPE) of ComBat-harmonized models (Philips/GE vendors). Validation cohort: Center2 (Philips, n = 103), Center3 (GE, n = 101); CE-T1WI/T1WI perform well, metrics consistent across vendors (abbreviations: GBM, PCNSL, CE, NE defined). [file 40644_2026_1018_MOESM4_ESM.pdf]

External validation performance of radiomics models stratified by device vendor

Sequence	Region	Vendor	AUC (95%CI)	ACC (%)	SEN (%)	SPE (%)
T2WI	CE region	Philips	0.831 (0.765-0.897)	75.2	83.5	72.4
		GE	0.805 (0.736-0.874)	73.8	82.0	70.9
	NE region	Philips	0.812 (0.743-0.881)	72.1	88.1	56.4
		GE	0.799 (0.728-0.864)	67.0	90.0	58.9
	Total tumor	Philips	0.845 (0.780-0.910)	76.2	84.0	68.8
		GE	0.837 (0.774-0.895)	72.8	84.0	68.8
T1WI	CE region	Philips	0.935 (0.892-0.978)	86.8	84.6	89.0
		GE	0.946 (0.871-0.995)	87.5	84.6	89.0
	NE region	Philips	0.788 (0.715-0.861)	73.4	86.0	65.7
		GE	0.773 (0.701-0.835)	71.1	86.0	65.7
	Total tumor	Philips	0.765 (0.686-0.844)	72.3	76.0	69.5
		GE	0.753 (0.672-0.829)	71.2	76.0	69.5
ADC	CE region	Philips	0.846 (0.782-0.910)	73.1	88.7	66.9
		GE	0.823 (0.755-0.891)	71.6	88.0	65.7
	NE region	Philips	0.815 (0.742-0.888)	76.4	86.0	71.4
		GE	0.800 (0.720-0.875)	75.3	86.0	71.4
	Total tumor	Philips	0.821 (0.748-0.894)	73.8	84.0	68.6
		GE	0.809 (0.731-0.883)	72.6	84.0	68.6
FLAIR	CE region	Philips	0.795 (0.723-0.867)	72.4	94.0	62.9
		GE	0.781 (0.712-0.843)	71.1	94.0	62.9
	NE region	Philips	0.762 (0.685-0.839)	71.9	84.0	65.9
		GE	0.749 (0.672-0.824)	70.7	84.0	65.9
	Total tumor	Philips	0.745 (0.666-0.824)	73.1	72.0	72.3
		GE	0.730 (0.654-0.816)	72.3	72.0	72.3
CE-T1WI	CE region	Philips	0.896 (0.865-0.959)	82.8	93.2	79.4
		GE	0.889 (0.819-0.933)	78.1	89.7	73.5
	NE region	Philips	0.875 (0.821-0.929)	78.6	92.7	72.9
		GE	0.843 (0.779-0.907)	75.3	90.2	70.1
	Total tumor	Philips	0.923 (0.881-0.965)	83.2	93.5	78.9
		GE	0.891 (0.837-0.945)	80.5	91.3	76.7
CT	CE region	Philips	0.815 (0.774-0.896)	77.1	87.5	72.6
		GE	0.807 (0.738-0.868)	73.9	84.3	68.7
	NE region	Philips	0.798 (0.729-0.867)	73.5	85.2	69.4
		GE	0.783 (0.710-0.856)	72.8	84.0	68.8
	Total tumor	Philips	0.821 (0.760-0.882)	75.6	85.9	70.4
		GE	0.801 (0.732-0.870)	73.3	84.1	68.5

Notes：All data were calculated based on radiomic features after ComBat harmonization to eliminate center-vendor batch effects；The external validation cohort consisted of Center 2 (Philips devices, n=103) and Center 3 (GE devices, n=101), with case distribution consistent with the actual clinical composition of each center.

GBM = glioblastoma; PCNSL = primary central nervous system lymphoma; CE = contrast-enhancing; NE = non-enhancing; ACC = accuracy; SEN = sensitivity; SPE = specificity.
